# Detecting head and neck squamous carcinoma using a portable handheld electronic nose

**DOI:** 10.1002/hed.26293

**Published:** 2020-06-03

**Authors:** Rens M. G. E. van de Goor, Michel R. A. van Hooren, Darius Henatsch, Bernd Kremer, Kenneth W. Kross

**Affiliations:** ^1^ Department of Otorhinolaryngology Head and Neck Surgery, Maastricht University Medical Center Maastricht The Netherlands; ^2^ Department of Otorhinolaryngology, Head and Neck Surgery Bernhoven Medical Center Uden The Netherlands

**Keywords:** diagnosis, electronic nose technology, head and neck squamous cell carcinoma, screening, volatile organic compounds

## Abstract

**Introduction:**

Detecting volatile organic compounds in exhaled breath enables the diagnosis of cancer. We investigated whether a handheld version of an electronic nose is able to discriminate between patients with head and neck squamous cell cancer (HNSCC) and healthy controls.

**Methods:**

Ninety‐one patients with HNSCC and 72 controls exhaled through an e‐nose. An artificial neural network based model was built to separate between HNSCC patients and healthy controls. Additionally, three models were created for separating between the oral, oropharyngeal, and glottic subsites respectively, and healthy controls.

**Results:**

The results showed a diagnostic accuracy of 72% at a sensitivity of 79%, specificity of 63%, and area under the curve (AUC) of 0.75. Results for the subsites showed an AUC of 0.85, 0.82, and 0.83 respectively for oral, oropharyngeal, and glottic HNSCC.

**Conclusion:**

This feasibility study showed that this portable noninvasive diagnostic tool can differentiate between HNSCC patients and healthy controls.

**Abbreviations**
ANN: artificial neural network
AUC: area under the curve
e‐nose: electronic nose
GC‐MS: gas chromatography‐mass spectrometry
HNSCC: head and neck squamous cell carcinoma
NPV: negative predictive value
PPV: positive predictive value
SE: sensitivity
SP: specificity
VOC: volatile organic compound

## 1 INTRODUCTION

Each year, more than 600 000 individuals are diagnosed with head and neck squamous cell carcinoma (HNSCC).^1^ These malignancies are associated with high morbidity and mortality rates.^2^ Two‐thirds of all HNSCC patients are diagnosed with advanced‐stage disease at first presentation. Long‐term survival rates for advanced HNSCCs are low and have not improved significantly over the last decades.^3^ Early diagnosis of HNSCC increases the likelihood of treatment with a single modality, lowers the risk of mortality, decreases medical expenditure, and improves patients' quality of life.^4^ For diagnosing HNSCC in an early stage, a reliable and cost‐effective screening instrument is required. Furthermore, for implementation in first‐line health care and rural areas, the instrument should be portable and easy‐to‐use. Currently, such a device is not available.

The electronic nose (e‐nose) could meet these requirements and be implemented to diagnose HNSCC in an early stage. E‐nose technology uses exhaled breath volatile organic compound (VOC) pattern analysis for classification. These VOCs are products of different metabolic processes, including cancer metabolism, that dissolve in the bloodstream and enter the respiratory tract through the alveoli.^5^, ^6^ Specific VOCs for HNSCC can be detected with e‐nose technology using pattern recognition in which nonspecific sensors are combined with machine learning techniques.^7^, ^8^, ^9^ An artificial neural network (ANN) can be trained to classify individual breath patterns resulting into a model for diagnosing head and neck cancer.

Previous studies by our group demonstrated that a laboratory e‐nose device operating with Tedlar bags is able to detect head and neck carcinomas, discriminating 36 smokers diagnosed with HNSCC from 26 healthy smokers, at a sensitivity of 90% and specificity of 80%.^8^ Using the portable e‐nose we found a diagnostic accuracy of 83% when differentiating between follow‐up patients with locoregional recurrent or second (or third) primary HNSCC and controls without evidence of disease.^10^ We also found that a portable handheld e‐nose (without a Tedlar/mylar bag or container) can distinguish between patients with lung cancer and a control group of healthy participants.^11^ Finally, we showed that e‐nose technology has the capability to discriminate between different types of cancers, including HNSCC, lung, bladder, and colon cancer.^8^, ^12^

In this follow‐up study, the capability of a portable and point‐of‐care handheld e‐nose was investigated to discriminate between HNSCC patients and a control group consisting of patients without a cancer history. If the e‐nose is shown to function in a larger population, this study might pave the way for routine use of the device as a diagnostic tool in a regular outpatient setting.

## 2 MATERIALS AND METHODS

### 2.1 Participants

This study was performed in a tertiary care referral hospital (Maastricht University Medical Center) from June 2013 to November 2017. Patients with pathohistologically confirmed glottic, oropharyngeal, or oral SCC were included as well as patients who visited the ear, nose, and throat (ENT) department for benign conditions, here referred to as healthy controls.

Exclusion criteria were age under 18 years, current tracheostomy, having had any treatment for a current tumor, and a history of any other sort of cancer. Furthermore, patients were excluded if they could not complete the full 5 minutes of measurement or if they were unable to endure a nose clip during measurement, which was used to promote oral breathing through the e‐nose. Their smoking habits and metabolic fasting state were documented. Non‐smoking was defined as no smoking in the previous month. Tumor characteristics and medical history were collected from the clinical records during regular visits at our outpatient department. Side‐effects or adverse events during or shortly after measurement were documented. The study protocol was approved by the medical ethics committee (PROTOCOL NUMBER 11407) and was designed in accordance with the declaration of Helsinki. Oral informed consent was obtained from all patients.

### 2.2 Materials

For this study we used four Aeonose devices (serial numbers 259, 309, 315, 362), using three micro hotplate metal‐oxide sensors (AS‐MLV sensors, Applied Sensors GmbH) and a Tenax tube. The combination of sensors and the Tenax tube ensures an optimal detection of the VOCs present, even at low concentration levels. The hotplates are periodically heated and cooled between 260°C and 340°C in 32 steps. During this process, exhaled air passes the sensors. The reduction and oxidation (redox) reactions of VOCs at the surface of the metal‐oxide sensors cause changes in conductivity of the sensors. The conductivity values recorded represent a unique exhaled‐breath pattern that can be analyzed.

### 2.3 Study design and participants

Both treatment and prognosis of HNSCC are dependent on the t‐stage and subsite of the tumor. The most common subsites of head and neck carcinomas are the oral cavity, oropharynx, and larynx. Based on these characteristics, four different models were created.^13^ The first included healthy controls that were compared to patients with HNSCCs of all subsites. Models 2, 3, and 4 consisted of healthy controls and patients with HNSCCs of the oral cavity, oropharynx, and glottis, respectively.

Before each measurement, patients were instructed to inhale and exhale into the e‐nose for 5 minutes through a disposable mouthpiece. This mouthpiece contains a high‐efficiency particulate arrestance (HEPA) filter, which protects the device to a large extent from contamination, for example, from bacteria and viruses. Patients were instructed to close their lips over the mouthpiece at all times, and a nose clip was used to prevent nasal air passage. Test runs of in‐ and exhalations were performed so the patient could get acquainted with the device. Participants breathed through a carbon filter to limit the possibility that environmental VOCs would tamper with the measurement. For the first 2 minutes, the lungs were rinsed with clean filtered air that passed through the carbon filter without passing the sensors, and dead air space was removed. Afterward, a valve was opened to ensure the passage of exhaled air over the sensors. The total measurement cycle lasted about 15 minutes, of which the patient in‐ and exhaled into the device for 5 minutes. The remaining time was used to measure any low‐concentrated VOCs inside the Tenax tube and to regenerate the sensors with clean filtered air (for details see van Hooren et al. 2016).^12^

Patients did not receive individual diagnostic results from the e‐nose analysis. The results from these measurements did not influence the regular diagnostic work‐up or treatment of the participants.

## 3 STATISTICAL ANALYSIS

Baseline group differences were determined using the independent sample *t* test, Fisher's exact test or Mann‐Whitney *U* test. All statistical analyses were performed using IBM SPSS Statistics for Windows, Version 25.0 (Armonk, NY: IBM Corp.).

During one measurement, 64 times 36 data points were recorded for each sensor. To compress these data points of temperature, measurement cycle and sensors, a Tucker3‐solution for tensor decomposition was used.^14^ The resulting vectors combined with the classification (benign of malignant) were used to train an Artificial Neural Network (ANN). Data compression and ANN have been integrated in a proprietary software package (Aethena—the eNose Company, Zutphen, the Netherlands). ANN training was executed for a number of data scaling options, resulting in multiple ANN options for separating between benign and malignant conditions. Data was cross‐validated using the Leave‐10%‐out method. This method prevents to a large extent the fitting of data on artifacts instead of breath‐profile classifiers.

We required at least five patients with HNSCC and five healthy controls to be measured per e‐nose device to eliminate possible device dependencies.

The ANN model calculates a value between −1 and 1 for each breath pattern, related to the diagnosis of that patient. For each model a threshold (range −1 to 1) was determined by the ANN to obtain the best possible diagnostic accuracy. Individual predicted values above this threshold were classified as positive, and values below this threshold were classified as negative. A Receiver‐Operator‐Curve is produced showing the performance of the model. By picking a position on this curve, a threshold is chosen for separating between positive and negative classified patients. In this way, data on sensitivity, specificity, area under the curve (AUC), and overall accuracy are obtained for each model.

## 4 RESULTS

### 4.1 Baseline characteristics

Between May 2013 and October 2017, 72 healthy controls and 91 patients with primary HNSCC originating from the oral cavity (37), oropharynx (34), and glottis (20) were included. Baseline characteristics, shown in Table 1, were comparable between both groups, except for “currently smoking,” which was significantly higher in patients with primary HNSCC.

**TABLE 1:** Baseline characteristics model 1

	Healthy controls	All subsites
Number of patients	72	91
Age (mean years)^a^	63	64 (*P* = .40)^a^
Sex (male)^b^	57	68 (*P* = .58)^b^
Currently smoking (yes)^b^	26	49 (*P* = .03)^b^
Pack years (mean)^c^	32	29 (*P* = .92)^a^
Tumor stage (n)		
1	\	16
2	\	22
3	\	12
4	\	41

a Independent *t*‐test.

b Fisher's exact test.

c Mann‐Whitney *U* test.

During this study, no adverse events were reported when using the Aeonose.

### 4.2 Data analysis

**FIGURE 1:**
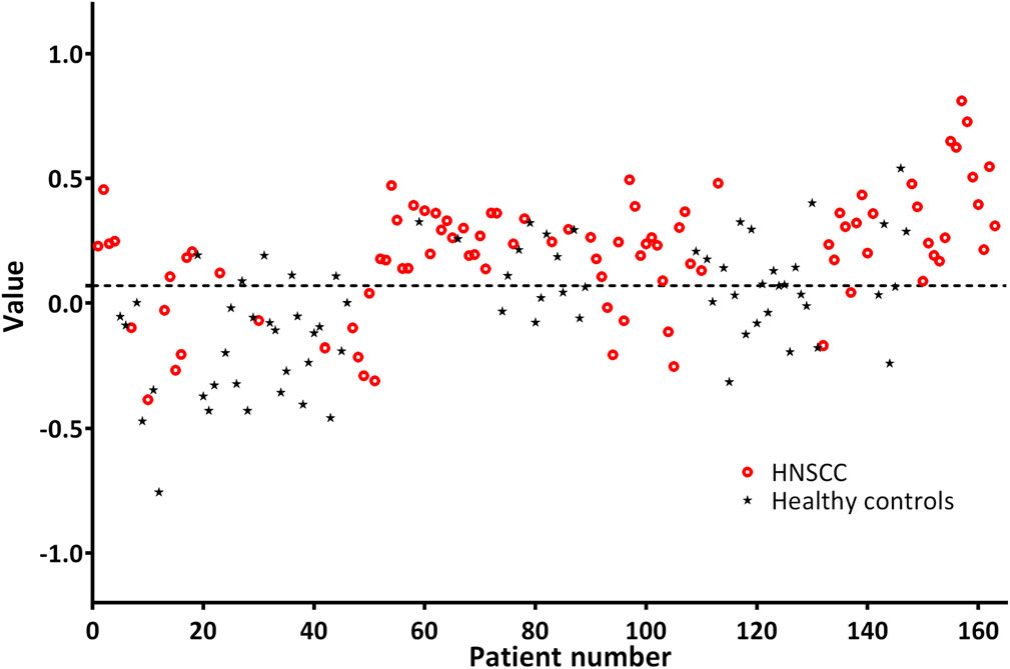
Healthy controls vs HNSCC of all subsites (model 1). The individual value of each patient and control calculated by the ANN is displayed. Values >0.07 are considered as positive for HNSCC. Red circles are patients with histopathologically confirmed HNSCC, and black asterisks represent healthy controls. ANN, artificial neural network; HNSCC, head and neck squamous cell cancer [Color figure can be viewed at wileyonlinelibrary.com]

The ANN for model 1 calculated a value between −1 and 1 for each patient and the threshold value for the highest diagnostic accuracy was calculated and set at 0.07. This means that a patient with a value below 0.07 was marked as negative for HNSCC and with a value above 0.07 as positive for HNSCC. In this way, 72 out of 91 HNSCC patients were correctly diagnosed with HNSCC, irrespective of tumor origin. Out of the 72 healthy controls, 45 were classified as healthy (Figure 1). This resulted in a diagnostic accuracy of 72%, sensitivity of 79%, and specificity 63% for model 1.

**FIGURE 2:**
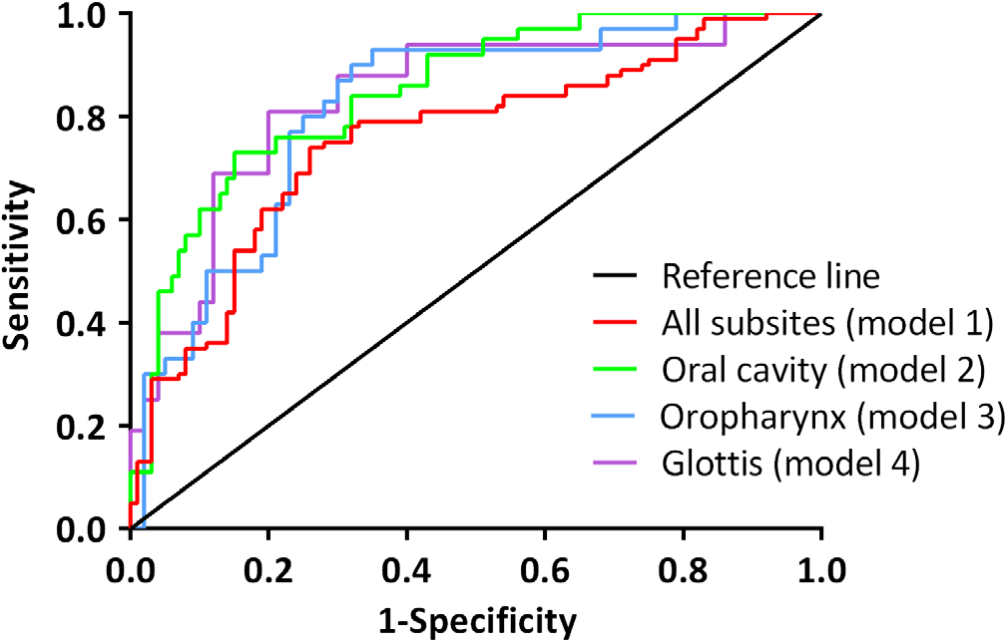
The ROC curve for each model. Black line represents the line of no‐discrimination [Color figure can be viewed at wileyonlinelibrary.com]

The thresholds for models 2, 3, and 4 were set at −0.07, −0.27, and −0.65, respectively. Analysis revealed that the sensitivity, specificity and AUC of the models 2 (oral cavity), 3 (oropharynx), and 4 (glottis) are all higher compared to model 1 (Figure 2).

The sensitivity, specificity, overall accuracy, and area under the curve of each model are shown in Table 2.

**TABLE 2:** The sensitivity, specificity, overall accuracy, and area under the curve of each model

	Model 1	Model 2	Model 3	Model 4
Sensitivity	79%	84%	87%	81%
Specificity	63%	67%	68%	76%
Accuracy	72%	72%	75%	77%
AUC	0.75	0.85	0.82	0.83

*Note:* model 1 (Healthy controls vs all HNSCC patients); model 2 (Healthy controls vs HNSCC subsite oral cavity); model 3 (Healthy controls vs HNSCC subsite oropharynx); model 4 (Healthy controls vs HNSCC subsite glottis).

Abbreviations: AUC, area under the curve; HNSCC, head and neck squamous cell cancer.

## 5 DISCUSSION AND CONCLUSION

In this study, we investigated the ability of the portable e‐nose to discriminate between patients diagnosed with HNSCC and healthy controls visiting our outpatient clinic for other benign diseases. We showed that the e‐nose is capable to distinguish between HNSCC patients, including all subsites, and healthy controls with a diagnostic accuracy of 72%. When analyzing the different subsites of HNSCC, the sensitivity, and specificity increases. This is probably due to the fact that the separate groups are more homogeneously than the combined one.

In recent years, investigations into the use of VOCs as potential biomarkers for head and neck cancer have drawn interest.^7^ Most of these studies have used gas chromatography‐mass spectrometry (GC‐MS), a technique that detects individual VOCs based on their molecular weight. The disadvantages of GC‐MS are the high costs, the need for specialized personnel to perform the analysis, and the need for an appropriate set of specific biomarkers for HNSCC. A variety of VOCs such as ethanol, 2‐propenenitrile and undecane dodecane, decanal, benzaldehyde, 3,7‐dimethyl undecane, 4,5‐dimethyl nonane, 1‐octene, and hexadecane have been described as potential biomarkers for the diagnosis of HNSCC.^7^, ^15^ Since GC‐MS relies on the detection of one single biomarker, this is a major limitation for the use of GC‐MS as a reliable screening instrument in the clinical setting. Bouza et al described elevated concentrations of benzaldehyde, 3,7‐dimethylundecane, and butyl acetate, measured by GC‐MS with Tedlar bags, and proposed considering these VOCs as potential biomarkers for oral SCC. Interestingly, they found that a higher concentration of butyl acetate was significantly correlated with a higher histological degree of differentiation. A disadvantage of their study is that, before sample collection, subjects were asked to abstain from food and drink (except water) and asked not to smoke in the 6 hours before sample collection.^15^ Garcia et al. reported 7 possible VOCs as biomarkers for laryngeal cancer: ethanol, 2‐butanone, 2,3‐butanediol, 9‐tetradecen‐1‐ol, octane derivative compound, cycloheptane derivative compound, and cyclo‐nonane derivative compound. In that study patients had to follow a strict protocol and were not allowed to eat, drink or smoke 8 hours prior to testing. The technique they used was a combination of solid phase micro‐extraction (SPME) with GC‐MS.^16^

Our results show that the e‐nose can discriminate between the different subsites of HNSCC, suggesting that each subsite of HNSCC has a different VOC profile. These differences between subsites have also been shown in a study by Gruber et al. Using an array of 6 nanomaterial‐based sensors combined with discriminant factor analysis (DFA), they found an accuracy of 83%, sensitivity of 77%, and specificity of 90% when comparing HNSCC patients with healthy controls. Even more interestingly, they found a sensitivity of 100% and specificity of 91% when comparing laryngeal squamous cell carcinoma with pharyngeal squamous cell carcinoma.^7^ A major difference with our study is that they used Tedlar bags for breath sampling and they did not allow the patients to eat, drink alcohol or smoke in the 12 hours prior to the measurement. That protocol might not be suitable for every patient visiting the care facility.

The e‐nose (Aeonose) as applied in this study is a handheld, fast, easy‐to‐use and portable device. In the future, the e‐nose might be incorporated in first‐line healthcare or used as a screening instrument, for instance in developing countries. We did not perform a special hygienic protocol that interrupted the daily routine of the patient, which could improve compliance. The device is able to connect to the internet via wifi and can run an unlimited number of validated models with only one measurement. Therefore, a patient at risk for HNSCC might be tested for glottic, oropharyngeal or oral cancer consecutively with models 2 to 4. Due to accurate temperature control of the sensors, these models can be easily transferred to an unlimited number of electronic noses. For the first time, mass application of electronic noses will be possible.^17^

## 6 CONCLUSION

We have shown that the e‐nose could be a promising diagnostic tool for detecting HNSCC, particularly when specific models are used for different subsites. An interesting application area could be in first‐line health care. Further investigation is warranted, notably of stage I and II tumors and larger groups of patients to allow modeling for each subsite.
